# Alien balsams, strawberries and their pollinators in a warmer world

**DOI:** 10.1186/s12870-021-03282-1

**Published:** 2021-10-30

**Authors:** Kamil Najberek, Andrzej Kosior, Wojciech Solarz

**Affiliations:** Institute of Nature Conservation, Polish Academy of Sciences, Al. Adama Mickiewicza 33, 31-120 Kraków, Poland

**Keywords:** Biological invasions, Crop yield, Alien plant control, Male bumblebee, Role of CHCs, Climate change

## Abstract

**Background:**

Strawberries are a common crop whose yield success depends on the availability of pollinators. Invasive alien plants, such as *Impatiens glandulifera* and *I. parviflora*, are also attractive for bees and hoverflies, respectively, and occur in close proximity to strawberry cultivation areas. The aim of the study was to test whether alien plants may decrease pollination of strawberry cultivation. However, even if the pollinators are abundant, efficiency of their pollination may decrease as a result of revisits of flowers that were already probed. It is addressed by pollinators by scent marking. Moreover, such revisits can be determined by nectar replenishment, which may occur rapidly in nectar-rich flowers. We studied revisits to *I. glandulifera* by bumblebees and defined the factors that influence the probability of revisits (air temperature; pollinator species; family caste and size; flower area; sun radiation; and time of day).

**Results:**

We found that the two alien species decreased the number of pollinators visiting strawberries. Apoidea, Bombini and Syrphidae significantly decreased on *Fragaria* × *ananassa* when alien *Impatiens* were present. We also revealed the influence of increasing air temperature on bumblebee foraging, which was particularly significant for female workers. At very high temperatures (> 37°C), bumblebee males revisited probed flowers less often than female workers.

**Conclusions:**

Our results demonstrate that in experimental conditions attractive alien species decrease pollination of strawberries, which may negatively affect production of this crop. Although the results have not been verified in real-life strawberry fields yet, we recommend that alien plant species that share the same pollinators and occur in close proximity of strawberries are controlled. Moreover, we found that revisits of probed flowers may weaken feeding efficiency of bumblebees. If revisits are not induced by nectar replenishment, then global warming may pose a serious threat to the survival of colonies, which may have consequences also for the plants that attract them, e.g., for strawberries.

**Supplementary Information:**

The online version contains supplementary material available at 10.1186/s12870-021-03282-1.

## Background

Pollination by wild insects is one of the key ecosystem services and plays an essential role in world crop production for human food [1]. Flowers of crops pollinated by wild pollinators produce twice as much fruit as flowers pollinated by the similar number of honey bees, *Apis mellifera* [2]. It is also known that in crop farms localized in areas with a high number of wild pollinators, the frequency of flower visitors is higher and the pollination process is enhanced [3, 4]. An example of such a crop is strawberry, *Fragaria* × *ananassa*, which is a very important and widely cultivated crop. In Spain alone, as many as 367 000 tons of strawberries per year are produced across 9 700 ha of fields [5]. Strawberries are valued for their taste and nutritional properties, including their antioxidant and anti-inflammatory effects on the nervous system [e.g., 6]. This species is mainly wind pollinated; however, if insect pollen transport does not occur, then the pollination rate rarely exceeds 60% [7, 8]. Moreover, for strawberry, the absence of pollinators decreases the fruit weight [7–10] and increases the malformation probability [9, 11, 12] while the presence of pollinators results in better fruit quality and increases total fruit production [8, 9, 13, 14].

Wild-growing invasive alien plant species have been shown to tempt pollinators away from native wild plants [e.g., 15]. An opposite scenario is also possible, because invasive alien plants growing in high-density populations could also act as “magnet” species that increase pollination of neighboring native plants [16]. However, these mechanisms have not been experimentally studied to date in cultivated plants. To bridge this gap, we selected two invasive alien plant species, Himalayan balsam (*Impatiens glandulifera*) and small balsam (*I. parviflora*) and tested whether they may decrease pollinators availability in cultivations of strawberry (*Fragaria* × *ananassa*) or conversely, enhance the pollination effect of this crop. The flowering periods of the three species overlap. Apidae (as in the case of *I. glandulifera*) and syrphidae (as in the case of *I. parviflora*) are common pollinators of *Fragaria* × *ananassa* [7, 17]. Therefore, it is likely that the two alien plants may decrease pollination of strawberries. In the study area (Małopolska, SE Poland), the two balsams occur regularly in close proximity to strawberry fields (Najberek, pers. observ.). It should also be stressed that *I. glandulifera* is a highly invasive alien species that is widely distributed throughout Europe while *I. parviflora* is invasive in central and northern parts of the continent [18]. *I. glandulifera* is the best nectar-rewarding plant in Europe (with nectar production of 0.3 mg per flower per hour), which probably determines its remarkable attractiveness for Apidae [15]. The production of nectar in flowers of *I. glandulifera* far exceeds its production in *I. parviflora* [19, 20]. On the other hand, the success of *I. parviflora* is driven mainly by its autonomous selfing ability [20]; in addition, its nectar is very attractive for many species of syrphidae [21–23].

The importance of plant-pollinator interactions for crops is associated not only with their diversity and numbers but also with their pollination efficiency [24]. Even if alien plant species do not decrease pollination of crops, the visitors of the flowers may be less efficient. The reason could be that a range of different factors, including weather conditions, may cause pollinators to more frequently revisit flowers that were already probed. In this regard, avoiding “empty” flowers that have been recently visited is of paramount importance not only for pollinators but also for crops. Any factor that decreases the effectiveness of pollinators represents a potential loss of the plant’s reproductive success [24]. It is known that bumblebees can detect their own scent marks secreted on visited flowers to avoid revisiting them during the foraging flight [25–29], and they also recognize flowers that have already been visited by their siblings, conspecifics or even by individuals from other bumblebee colonies. An underlying mechanism is that while pollinating, bumblebees secrete a mixture of hydrocarbons from their glandular systems associated with the claw retractor tendon of legs [30]. The process is essential for bumblebee colony economy because it saves time probing empty flowers and consequently results in an increase in the efficiency of pollen and nectar collection [25–29]. This efficiency is mostly affected by the choice of flower; therefore, incorrect decision-making may have negative consequences for bumblebees as well as for plants that attract them to transfer their pollen [24]. On the other hand, examples have been observed when revisits of flowers could play a positive role and should not be considered incorrect decisions. A previous study demonstrated [31] that some plants can replenish nectar within minutes, which may attract a pollinator even during the same foraging flight. This replenishment may be determined by nectar removal and supported by the airborne sound of the pollinator [32], its touch [33] and its vibration [34]. Considering the extraordinary abilities in nectar production of highly invasive alien plants (such as *I. glandulifera* [15]), the role of nectar replenishment in plant-pollinator interactions can be significant.

We defined factors that influence the probability of revisits of probed flowers of *I. glandulifera* by their bumblebee pollinators, with the main focus on air temperature. Although the negative role of increasing air temperature on plant-pollinator interactions has been broadly discussed [35–39], knowledge of the impact of temperature on scent-marking is still limited. To date, temperature has been shown to alter the efficacy of sexual chemical signals in the mountain lizard *Iberolacerta cyreni* [40] and female oviposition behaviour and the perception of infochemicals emitted by larvae of the ladybeetle *Adalia bipunctata* [41]. In turn, although the impact of increasing temperature on nectar replenishment was not investigated using *Impatiens* species, investigations using other plants showed that moderately elevated temperatures may facilitate nectar secretion while very high temperatures reduce nectar secretion [e.g., 42, 43]. Time of the day was also included in the study design because the activity of pollinators of *Impatiens* species may change as the day progresses [44, 45], which could be associated with diurnal changes in the amount of nectar. Another factor was the level of sun radiation because light intensity can constrain foraging efficiency [46]. Moreover, the size of visited flowers and the caste of pollinators were taken into account because they may affect both the search time of initially visited flowers and the flight behaviour of foraging bumblebees [47–49].

In this study we conducted two experiments to address two research questions. Firstly, whether wild-growing invasive alien plant species decrease pollination of cultivated plants, or conversely, they act as magnet species and increase the pollination rate of crops. Secondly, whether the increasing number of revisits of flowers that were already probed (lowering efficiency of pollination) is influenced by the weather, including rising air temperature.

## Results

### Experiment 1: Invasive alien species decrease pollination of cultivated species

**Fig. 1:**
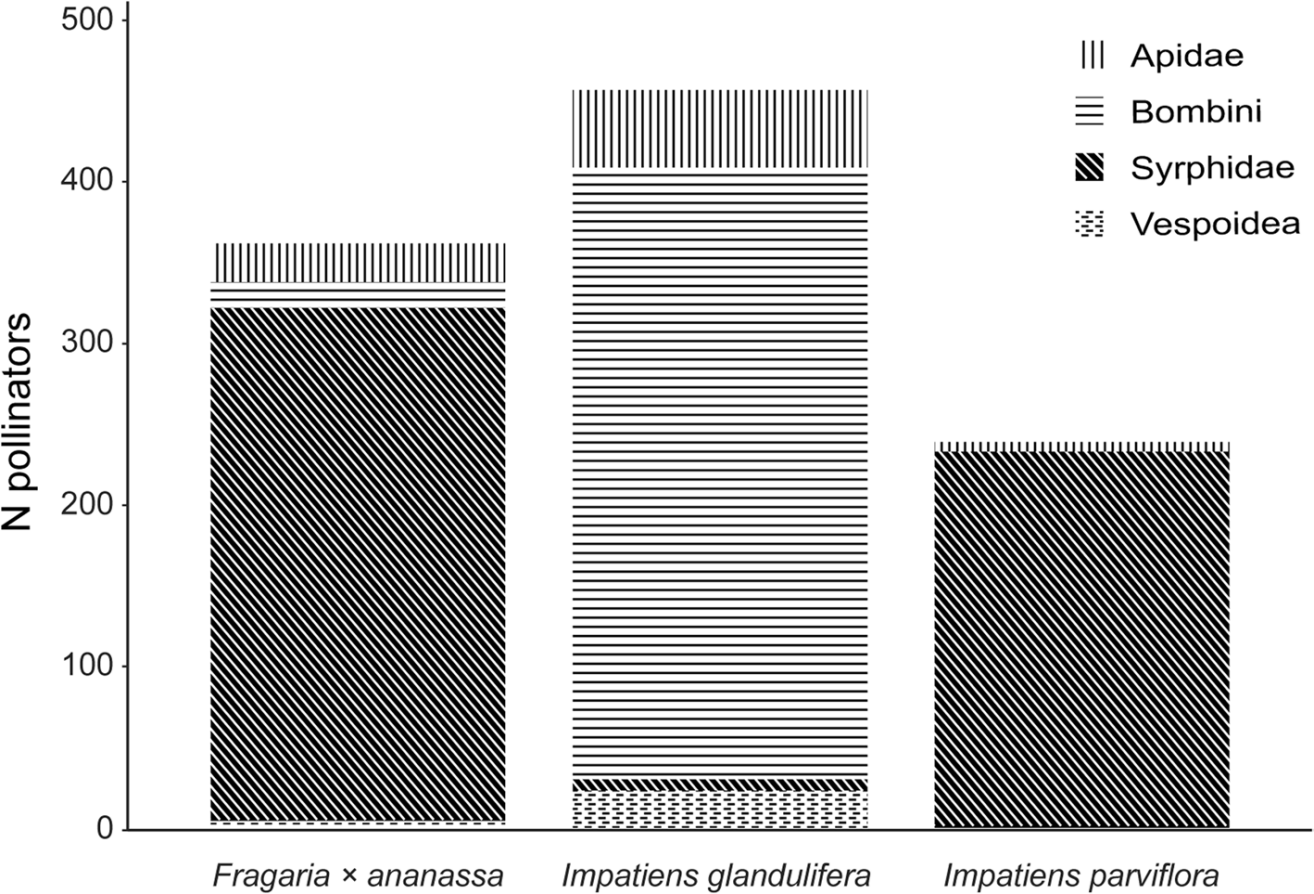
Major pollinator groups recorded on cultivated *Fragaria* × *ananassa*, highly invasive alien *Impatiens glandulifera* and invasive alien *I. parviflora*. The detailed list of recorded pollinators is included in Table S1 (Online Resource). All insects that had contact with flower anthers or stigmas were considered pollinators

In total, we recorded 3241 pollinators (Online Resource, Table S1), and 27.0% were identified at the species level while 35.8% were identified at the family level. In addition, 36.6% belonged to the *Bombus lucorum complex*, and few records (0.5% in total) were attributed to superfamilies, genera and tribes. The dominant pollinator groups of *I. parviflora* and *Fragaria* × *ananassa* were different from those of *I. glandulifera* (Fig. 1; Online Resource, Table S1). In the two former species, the dominant group was Syrphidae (e.g., *Episyrphus balteatus* and *E. corolla*; Fig. 1; Online Resource, Table S1), while in *I. glandulifera,* the dominant group was Bombini (*B. lucorum-complex*, *Bombus pascuorum*, *B. terrestris* and *B. hypnorum*; Fig. 1; Online Resource, Table S1). Interestingly, although bumblebees were not very abundant on *Fragaria × ananassa* (Fig. 1; Online Resource, Table S1), as many as 6 bumblebee species visited their flowers (*B. pascuorum*, *B. lucorum-complex*, *B. terrestris*, *B. humilis*, *B. pratorum* and *Psithyrus vestalis*; Online Resource, Table S1). The share of Apidae pollinators was also substantial (Fig. 1). For example, *A. mellifera* was an important pollinator of *I. glandulifera* and also occurred on *Fragaria × ananassa* (Online Resource, Table S1). Formicidae, which is known to include nectar thieves, occurred on *Fragaria × ananassa* and occasionally on *I. parviflora* (Online Resource, Table S1). It should also be noted that when the cultivated plant (*Fragaria × ananassa*, “Fr”) was exposed together with the alien plant (*I. glandulifera* or *I. parviflora*, “Im”), Variant 3 (FrIm), only a small number of recorded pollinators visited both surveyed plant species during a single flight (N = 35, 1.1%; including 33 Syrphidae and 2 Diptera taxa). All those cases were recorded only in the tests of strawberries and *I. parviflora*.

**Fig. 2:**
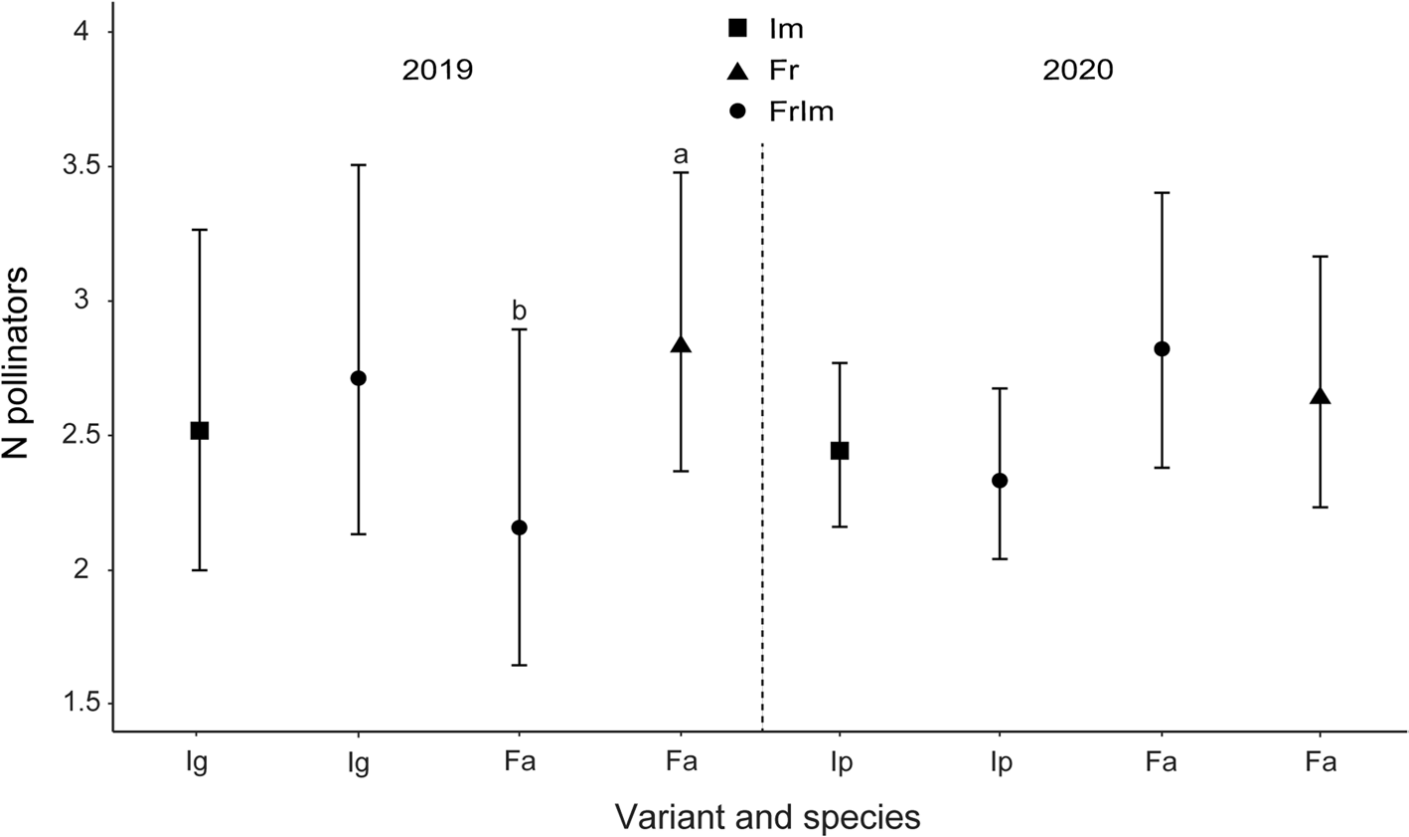
Estimated mean number of pollinators (± confidence intervals) recorded from highly invasive alien *Impatiens glandulifera* – ‘Ig’, invasive alien *I. parviflora* – ‘Ip’ and cultivated *Fragaria* × *ananassa* – ‘Fa’ in the three study variants: Im (alien species exposed solely), Fr (cultivated species exposed solely) and FrIm (alien and cultivated species exposed together). Letters above the T-bars indicate a significant decrease in the number of recorded pollinators from *Fragaria* × *ananassa* for tests carried out without *I. glandulifera* (letter “a”) and with *I. glandulifera* (letter “b”)

In statistical analysis the GLMM model with two fixed effects (‘Variant and species’, ‘Stem height’) and two random factors (‘Plant ID’, ‘Time intervals’) was selected (Table S2). In the model, ‘Variant and species’ variable played a crucial role (F = 2.04, df = 1420, *p* = 0.047). We revealed that the number of pollinators of *Fragaria* × *ananassa* was significantly higher when the species was exposed alone (Fr) than when it was exposed together with the highly invasive *I. glandulifera* (FrIm) (contrast: SE = 0.35, t = -1.95, df = 1413, *p* = 0.052; Fig. 2). In contrast, the number of pollinators of *I. glandulifera* tended to rise when the two species were exposed together (FrIm) (contrast: SE = 0.10, t = 1.81, df = 1413, *p* = 0.070; Fig. 2).

In addition, we compared the number of records of Bombini, Syrphidae, Apoidea and Vespoidea in particular plant arrangement variants (Im, Fr and FrIm) using proportion tests (Table 1). We found that the total numbers of Bombini and Syrphidae recorded on *Fragaria* × *ananassa* were significantly higher when it was the only species available (Fr; Table 1) than when it was accompanied by *I. glandulifera* (FrIm; Table 1). At the same time, we found that the number of Bombini recorded from the latter species was significantly lower in the Im variant than in tests together with *Fragaria* × *ananassa* (FrIm) (Table 1). Interestingly, the number of Apoidea visiting *I. glandulifera* solely (Im) was higher than in the tests with *Fragaria* × *ananassa* (FrIm) (Table 1).

**Table 1:** Number of pollinators per taxon group, recorded from highly invasive alien *Impatiens glandulifera* – ‘Ig’, invasive alien *I. parviflora* – ‘Ip’ and cultivated *Fragaria* × *ananassa* – ‘Fa’ in the three study variants: Im (alien species exposed solely), Fr (cultivated species exposed solely) and FrIm (alien and cultivated species exposed together). All insects that had contact with flower anthers or stigmas were considered pollinators

Plant species	Species combination	Variant	Pollinator group	N records	Proportion test
*Fragaria × ananassa*	Fa	Fr	Apoidea	17	**Fa** vs **Fa/Ip**: χ2 = 28.73, *p* < 0.001
	Fa / Ip	FrIm	Apoidea	2	
	Fa	Fr	Bombini	19	**Fa** vs **Fa/Ip**: χ2 = 28.73, *p* < 0.001 **Fa** vs **Fa/Ig**: χ2 = 32.68, *p* < 0.001
	Fa / Ip	FrIm	Bombini	5	
	Fa / Ig	FrIm	Bombini	2	
	Fa	Fr	Syrphidae	320	**Fa** vs **Fa/Ip**: χ2 = 61.03, *p* < 0.001 **Fa** vs **Fa/Ig**: χ2 = 581.79, *p* < 0.001
	Fa / Ip	FrIm	Syrphidae	289	
	Fa / Ig	FrIm	Syrphidae	21	
	Fa	Fr	Vespoidea	1	NS
	Fa / Ig	FrIm	Vespoidea	1	
*Impatiens glandulifera*	Ig	Im	Apoidea	85	**Ig** vs **Ig/Fa**: χ2 = 93.36, *p* < 0.001
	Ig / Fa	FrIm	Apoidea	35	
	Ig	Im	Bombini	836	**Ig** vs **Ig/Fa**: χ2 = 386.18, *p* < 0.001
	Ig / Fa	FrIm	Bombini	1035	
	Ig	Im	Syrphidae	1	NS
	Ig / Fa	FrIm	Syrphidae	3	
	Ig	Im	Vespoidea	11	NS
	Ig / Fa	FrIm	Vespoidea	15	
*Impatiens parviflora*	Ip	Im	Apoidea	4	NS
	Ip / Fa	FrIm	Apoidea	2	
	Ip	Im	Syrphidae	234	**Ip** vs **Ip/Fa**: χ2 = 106.09, *p* < 0.001
	Ip / Fa	FrIm	Syrphidae	179	

Although the overall result of the GLMM model for *Fragaria* × *ananassa* and *I. parviflora* did not reveal any between-species differences (Fig. 2), such differences were found in the proportion tests (Table 1). The numbers of Apoidea, Bombini and Syrphidae recorded from *Fragaria* × *ananassa* were significantly lower with (FrIm) than without (Fr) the alien species (Table 1). However, it should also be noted that the number of Syrphidae recorded from *I. parviflora* in the FrIm variant with *Fragaria* × *ananassa* also decreased (Table 1).

The GLMM model also revealed that taller plants were more frequently visited by pollinators than shorter plants (F = 16.73, df = 1414, *p* < 0.001). The number of flowers and air temperature did not influence the results (Online Resource, Table S2). Datasets on the stem height of surveyed plant individuals are included in Table S3 (Online Resource).

### Experiment 2: Revisiting flowers increases with air temperature

**Fig. 3:**
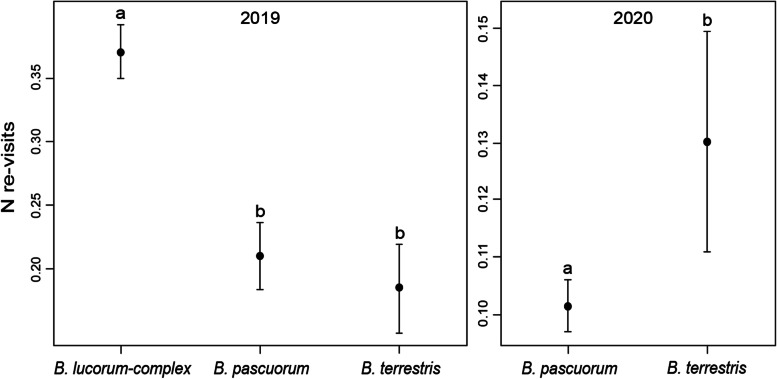
Mean number of revisits (±SE) to *Impatiens glandulifera* flowers for *Bombus lucorum-complex*, *B. pascuorum* and *B. terrestris* in the two study years, 2019 and 2020 (see Table 2). Letters above the T-bars indicate significant differences between the bumblebee species

In 2019, revisits of *I. glandulifera* flowers by bumblebees amounted to 24.4% of all visits (total N of visited flowers = 1434) paid during 195 flights. During a single bumblebee flight, 278 flowers had 2 revisits (19.4%), 61 had 3 revisits (4.25%), 10 had 4 revisits (0.7%) and one had 5 revisits (0.07%). We recorded flights of the *B. lucorum complex* (*N* = 105), *B. pascuorum* (*N* = 49) and *B. terrestris* (*N* = 41). The GLMM model with three fixed effects (‘Temperature’, ‘Pollinator’, ‘Cloud cover’) and one random factor (‘Plant ID’) was selected (Table S2). The result was affected by air temperature and bumblebee species (Table 2). The same flowers were revisited more frequently when the air temperature was higher (Table 2; Online Resource, Fig. S1). A similar tendency was also found in sunny weather in comparison to partially sunny weather (Table 2; Online Resource, Fig. S2); in turn, in cloudy weather the results did no differ from both sunny/partially sunny weather (Table 2; Online Resource, Fig. S2). Moreover, the number of revisits differed among the *B. lucorum complex* (*N* = 112), *B. pascuorum* (*N* = 52) and *B. terrestris* (*N* = 43), with individuals of the former species revisiting the same flowers more frequently than the two latter species (differences at *p* < 0.001; Fig. 3/2019). Interaction between temperature and pollinator species was nonsignificant in the model (Online Resource, Table S2).

**Table 2:** glmmADMB best-fit models for the revisits of flowers of *Impatiens glandulifera* by bumblebees in 2019 and 2020. Air temperature and pollinator species were included in the two models. The 2019 model included also cloud cover, while the 2020 model – the time of day, pollinator gender (male/female worker), area of flower profiles and interaction between the air temperature and pollinator gender

Year	Effect	χ2	df	*p*
2019	Temperature	27.55	1	< 0.001
	Pollinator	28.67	2	< 0.001
	Cloud cover	5.72	2	0.057
2020	Temperature	22.66	1	< 0.001
	Day time	3.01	2	0.2
	Pollinator	19.44	1	< 0.001
	Gender	48.7	1	< 0.001
	Flower area	5.22	1	0.02
	Temperature*Gender	34.9	1	< 0.001

**Fig. 4:**
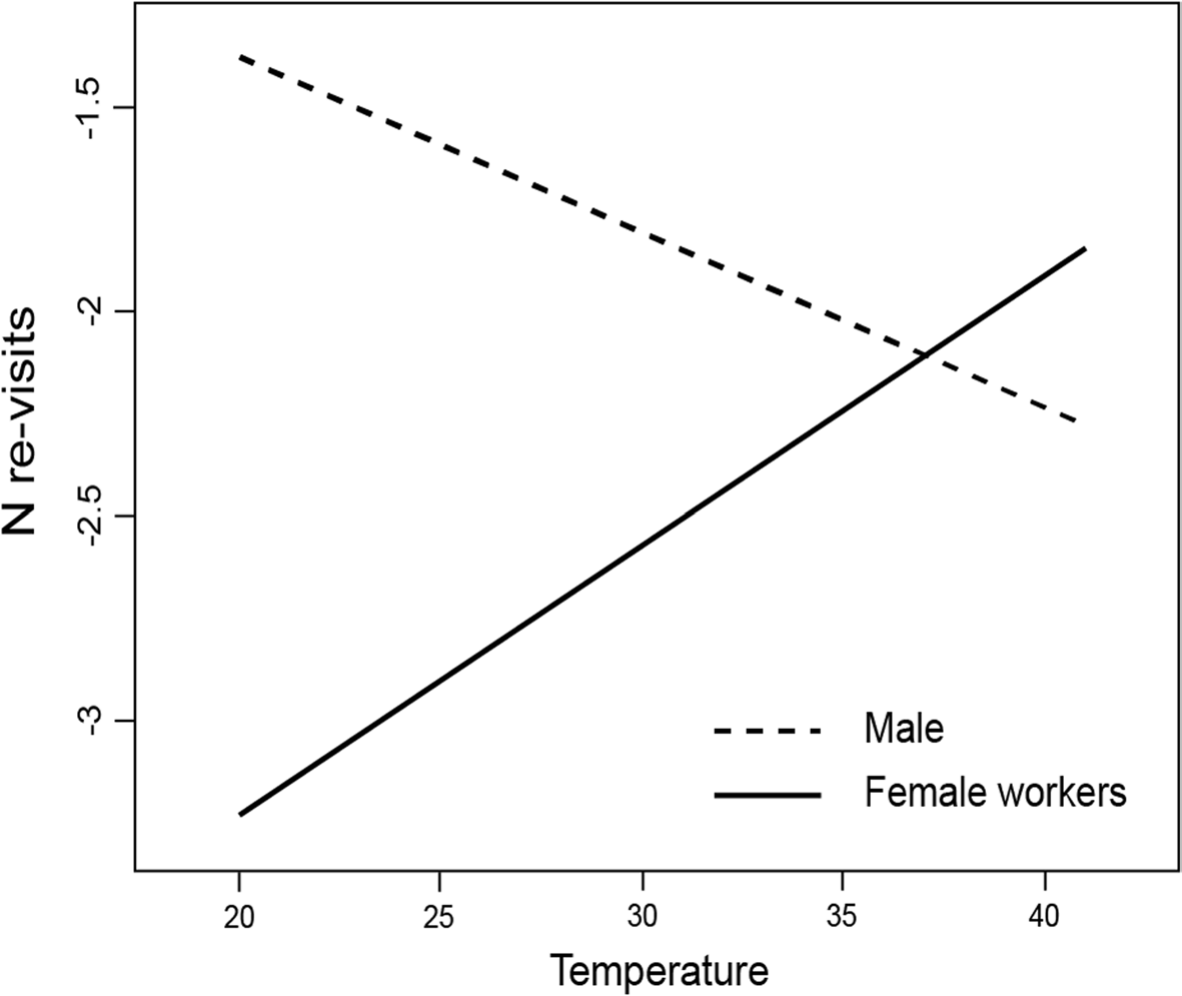
Interaction plot between air temperature and pollinator gender (male/female worker) in the glmmADMB model with the estimated number of revisits of *Impatiens glandulifera* flowers as a target variable (see Table 2). The values on y-axis are in glmmADMB procedure default link-scale (log)

In 2020, 9.7% of the flowers were revisited (total N of visited flowers = 5679; N flights = 484), and during a single bumblebee flight, 515 flowers had 2 revisits (9.07%), 33 had 3 revisits (0.58%), 10 had 1 revisit (0.18%) and one had 5 revisits (0.02%). We recorded the flights of 86 males (*B. pascuorum*), 394 female workers (348 *B. pascuorum*, 44 *B. terrestris*, 2 *B. hypnorum*) and 4 queens (3 *B. hypnorum*, 1 *B. terrestris*). The GLMM model with five fixed effects (‘Temperature’, ‘Day time’, ‘Pollinator’, ‘Gender’, ‘Flower area’), one interaction (‘Temperature*Gender’) and one random factor (‘Plant ID’) was selected (Table S2). As in 2019 (Online Resource, Fig. S1), we confirmed that the revisits significantly increased with air temperature (Table 2). Moreover, in general, males revisited flowers more frequently than female workers (Table 2). However, the interaction ‘Temperature*Gender’ revealed that at a very high temperature (exceeding 37°C), males make fewer revisits than female workers (Table 2, Fig. 4). At lower temperatures, the opposite trend was observed, with fewer revisits by female workers (Fig. 4). However, the only recorded male was *B. pascuorum*; thus, we were not able to check whether males of other species presented similar reactions to high temperatures. In turn, workers of *B. hypnorum* and all queens were too rare to be included in the results analysis. Between-species differences were also significant in that *B. terrestris* revisited the flowers more frequently than *B. pascuorum* (Table 2; Fig. 3/2020). The model also showed that larger flowers were revisited more often than smaller flowers (Table 2). Time of day, bumblebee size, sun radiation or the two interactions (temperature with pollinator species and temperature with pollinator size) were all nonsignificant in the model (Table 2; Online Resource, Table S2).

In 2020, we also tested factors that may determine which plants are most frequently chosen as the first to be visited. Stem height, size of flowers or their hue, were not important in this respect (Online Resource, Table S4); datasets on stem height and flower size of *I. glandulifera* individuals are included in Table S3 (Online Resource). Moreover, although GIS spatial analysis of the distribution of plants that were first to be visited in 2020 suggested that they were usually situated close to the plot edge (Online Resource, Fig. S3), the non-significant result for ‘Location’ variable in the GLM model did not supported this conclusion (Online Resource, Table S4).

## Discussion

### Invasive alien species decrease pollination of cultivated species

*Impatiens glandulifera* and *I. parviflora* are invasive alien species with a negative impact on nature conservation and human economies [15, 50, 51], while *Fragaria* × *ananassa* is a common, flavourful and healthy crop [6, 13]. We revealed that the two alien species may compete with the cultivated species for pollinators. Previous studies [8, 9, 11–14] demonstrated that the availability of pollinators determines the quality and volume of strawberry production. The results of our study, however, were obtained from cultivation plot experiments with a similar number of flowering plants per species. Their arrangement on the plot and the fact that there were approximately twice as many flowers in the variant in which alien and cultivated species were exposed together then in single-species variants, did not reflect real life conditions. These limitations may have synergistically affected the obtained results. It may be also important that the experimental *Impatiens* had significantly less flowers than wild-growing plants. For instance, experimental *I. glandulifera* had only 1-8 flowers per individual (average = 2, *N* = 45), while those from Kraków, Muszyna and Izerskie foothills had 4-138 flowers per individual (average = 34, *N* = 90; Najberek, unpubl. data). It suggests that under natural conditions the decrease in crop pollination can be significantly higher than it was demonstrated in this study. Thus, our outcomes should be verified using real-life strawberry crops with neighbouring *Impatiens*. Natural mosaics of crops and *Impatiens* patches may differ in area, density of individuals, with differing number of flowers and plant array, which may also play an important role in the studied plant-pollinator interactions. Real-life strawberry fields would allow assessment of the relevance of pollinator activity reduction on yield.

In the two study years, we demonstrated that the presence of the highly invasive alien *I. glandulifera* or less invasive *I. parviflora* decreased the number of pollinators that visited *Fragaria* × *ananassa*, with the most pronounced differences in the comparisons with *I. glandulifera*. This result is opposite to the assumption that the two *Impatiens* species may act as magnet species and enhance the pollination of strawberries. The GLMM showed that the overall number of pollinators of *Fragaria* × *ananassa* decreased when *I. glandulifera* was present, and the proportion tests indicated that the taxa associated with this decrease were Bombini and Syrphidae. The former group rarely visited *Fragaria* × *ananassa*; however, hoverflies were the main pollinators of this crop in our study.

In the comparison between *Fragaria* × *ananassa* and the less invasive alien *I. parviflora,* the GLMM model did not reveal any differences. However, the proportion tests indicated that Apoidea, Bombini and Syrphidae significantly decreased on *Fragaria* × *ananassa* when *I. parviflora* was present. This finding suggests that *I. parviflora* may decrease pollination of strawberry, which is similar to that observed for *I. glandulifera*.

Interestingly, the number of Apoidea recorded from *I. glandulifera* was higher in tests conducted solely for this species than when it was exposed on the plot with *Fragaria* × *ananassa*. A similar situation was noted in the case of Syrphidae of *I. parviflora*. These results indicate that alien species may also be negatively affected when they co-occur with strawberry crops. The impact of the decrease in Apoidea on *I. glandulifera* was probably negligible because in our plot, it was pollinated mainly by bumblebees. However, it potentially would play a more significant role in areas where bees constitute the dominant pollinator group. In turn, Syrphidae are the main pollinators of *I. parviflora* [21–23], and their absence may reduce the spread of this plant. A high number of flowers in a habitat patch has been found to positively influence the number of hoverflies [52]. Although the combination of *I. parviflora* and *Fragaria* × *ananassa* provided at least twice as many flowers as each of the single plant species variants, the number of Syrphidae decreased. Therefore, some other factors (stronger than flower abundance) impeded the presence of Syrphidae in our experiment. *I. parviflora* individuals were twice as tall as *Fragaria* × *ananassa* individuals; thus, flowers of the former species were more available than the flowers of the latter. Hoverflies adjust their flight altitude to be optimal to detect pollen and nectar based on optical and olfactory cues [53], and the co-occurrence of the two species that significantly vary in height may be confusing for hoverflies. In addition, *I. parviflora* and *Fragaria* × *ananassa* differ in terms of flower size, and these characteristics may also determine the flight altitude [48]. Flowers of the latter species are larger and could be detected earlier and at higher altitudes. It should also be noted that the presented tests were conducted using an experimental design that included the random arrangement of strawberries and the two balsam species in plots in very close proximity to each other. This dependence could be modified to test real-life crop fields and neighbouring alien plant patches. The impact of alien species should be stronger in close proximity to the field and weaker in more distant plots. However, further tests are needed to confirm this assumption.

The dependence between flight altitude and plant height should also be considered regarding the results of the GLMM analysis. The model revealed that taller plants are more frequently pollinated than shorter plants. The tallest species included in the study was *I. glandulifera,* which is very attractive to pollinators [15]. However, the attractiveness of *I. glandulifera* did not play a only role because individuals of this species were also very diverse in terms of their height (65-182 cm). This result, therefore, also demonstrates that pollinators detect flowers of taller plants quicker than flowers of shorter plants. Moreover, in the context of biological invasions, it can be assumed that tall alien plants, including the highly invasive alien *I. glandulifera* [15], may be very effective in decreasing pollination of native plants.

We experimentally demonstrated that alien plants may decrease the pollination of crops. Although not directly tested during the present study, it was proved that lower pollination by insects usually negatively affects crop production [e.g., 14, 54]. A practical implication is that alien plant species that have common pollinators with a given crop and occur in close proximity to the cultivation area should be controlled. This pertains not only to *I. glandulifera* but also to many other alien plants attractive for pollinators (e.g., *I. capensis*, *I. balfourii*, *Asclepias syriaca*, *Solidago gigantea*, *S. canadensis*, *Reynoutria japonica*, *Spiraea tomentosa*). Although the ability of these alien species to tempt pollinators away from crops has not been directly tested, their control is recommended near crops.

A buffer zone in which to undertake control measures should be individually set out for particular crop types according to the migration capability of their pollinators. For example, tomatoes, cucumbers, muskmelons, watermelons, raspberries, black currant, lavender, bilberry, cherry trees, peach trees or apricot trees are pollinated by bumblebees that can collect nectar and pollen from preferred food sources situated as far as 1500 m away from their colonies [55]. Therefore, alien plants should optimally be controlled within a 1500 m buffer from those crops. On the other hand, the migration capabilities of Syrphidae are more limited than those of bumblebees and can cover distances up to 200 m from the food source [53]. Therefore, a 200 m control buffer around *Fragaria* × *ananassa* crops seems appropriate. In addition, it should be also noted that the localities of wild-growing *I. parviflora* and *I. glandulifera* occurred 400 and 4700 meters, respectively, from the cultivation plot. Considering the migration capability of hoverflies, bumblebees and bees [53, 55, 56] those localities did not influence the obtained results.

### Revisiting of flowers increases with air temperature

Bumblebees secrete a mixture of hydrocarbons from their leg tendon glands [30] that can be recognized by themselves as well as their siblings and conspecifics during foraging flights [25–29]. The efficiency of nectar feeding of colony conspecifics is mostly affected by flower choices; therefore, revisits to probed flowers may have negative consequences for the colony economy as well as for the pollination rate of host plants [24]. In our 2-year study, revisits and flower choice were studied for three bumblebee species, *Bombus pascuorum*, *B. lucorum complex, B. terrestris*, and an alien plant, *Impatiens glandulifera*, which is known to produce high amounts of nectar. Interestingly, we found that the number of revisits increased with the air temperature. It is known that climate warming may lead to morphological, physiological and functional mismatches between plants and pollinators [35–39, 57–60] as well as to a lower availability of food plants [61–63]. The negative impact of increasing temperature on the effectiveness of scent marking, however, has only been studied occasionally and not in the context of plant-pollinator interactions [40, 41].

The increase in the number of revisits with air temperature was particularly significant for female workers. In general, female *B. pascuorum*, *B. lucorum-complex,* and *B. terrestris* revisited probed flowers less frequently than males (*B. pascuorum*). However, at very high temperatures (> 37°C), the opposite result was observed, with more revisits by female workers. These trends may be associated with differences in the composition of cuticular hydrocarbons (CHCs) secreted by bumblebees between males and females. Males secrete significantly more compounds with longer chain lengths (identified as ‘wax esters’) than female workers and queens [30]. At the same time, CHCs with longer-chain compounds have higher melting temperatures and insects equipped with such a waxy layer efficiently manage water [64, 65]. Therefore, it can be assumed that the body of bumblebee males is better protected against extremely high temperatures, and these better conditions of males may, in turn, result in a lower number of errors in flower choice. Another role of CHCs is to facilitate communication between insects by conveying various types of information [64, 65]. For example, it is known that CHC male compounds allow them to mark a flight route to attract virgin queens. We assume that scent marks created using longer-chain wax esters also have other functions and may be more durable at hot temperatures than scent marks secreted by female workers. This presumption is worthy of experimental testing in the future.

However, it should be stressed that we did not assess the nectar content in the visited flowers because the assumption was that bumblebees would avoid previously probed and scent-marked flowers during a single flight regardless of the production potential of the plant. Moreover, it is well known that *I. glandulifera* produces significantly more nectar than *Fragaria* × *ananassa* [15, 19]. A disadvantage of this approach was that we did not test whether nectar replenishment in probed flowers may occur during a single foraging flight. Previous studies demonstrated that replenishment occurs rapidly, i.e., within minutes [31]. For instance, replenishment was found to occur within 3 minutes based on pollinator sounds [32]. In the present study, a single bumblebee flight took only approximately 45 seconds, whereas the number of re-visits per single flight was between 1 and 5. However, as *I. glandulifera* is the best nectar rewarding plant species in Europe [15], it cannot be excluded that nectar replenishment occurs more rapidly, i.e., within seconds. If *I. glandulifera* was capable of such remarkably fast replenishment, then the meaning of the recorded revisits during the tests would be changed. Such changes would mean that at very high temperatures, workers can better recognize nectar-replenished flowers than males, and it would also mean that males are more efficient at temperatures below 37°C. If this supposition holds true, then the role of males in colony success could be even higher than that under the assumption that revisits are erroneous choices. Nevertheless, further studies on bumblebee revisits to Himalayan balsam are needed to provide further insights.

A previous study [48] demonstrated that large flowers are easier to find for bumblebees, which reduces the flight time. In contrast, we found that the number of revisits increased with the flower size. Larger flowers produce higher volumes of nectar than smaller flowers [66]; therefore, more intensive olfactory cues of larger flowers may impede the recognition of scent-marks secreted by bumblebees. It is likely that such cues could play an important role in the case of nectar-rich flowers of *I. glandulifera*; however, neither nectar volume nor sugar concentration were assessed in this study. Thus, this aspect needs to be better investigated, and our results should be considered with due caution.

In Experiment 2, we also indicated *I. glandulifera* individuals that likely were the first to be visited by newly arriving bumblebees. The graphical GIS analysis of the arrangement of surveyed plants in particular surveys suggested that the plants close to the edge of the plot, were most likely to be detected first. Interestingly, previous studies demonstrated that higher diversity and numbers of Syrphidae pollinators were recorded in field margins than in within-crop wildflower patches, despite the lower flower density in the former patches [52]. This finding was explained by the fact that field margins may offer additional rewards other than pollen and nectar availability (such as a high abundance of aphids for aphid-predatory insects) [52]. However, our finding was not supported by the GLM results in any respect. The stem height, flower size, flower hue also did not play a role in this analysis. It should, however, be noted that our study was conducted at experimental plot, thus it cannot be excluded that in real-life strawberry field tests the results would be more pronounced.

## Conclusions

Alien plant species are often distributed in close proximity to cultivation fields; however, their influence on crop yields has not been experimentally tested to date. In the present study, we demonstrated that two invasive alien species, *I. glandulifera* and *I. parviflora,* may decrease pollination of strawberry, *Fragaria* × *ananassa*. The results were obtained under experimental conditions that included a similar number of flowering plants per species and a random plant arrangement, although they did not include real distances between the crop and patches of alien balsams, and real number of flowers per species that are available for pollinators in natural conditions. Therefore, further tests in real-life strawberry fields and neighbouring patches of balsams are recommended to confirm that reduction in pollinator activity on strawberries is actually relevant.

Flower choice decisions may have consequences for both pollinating bumblebees and the plants that attract them. In this study, we revealed that the revisits of probed flowers of *I. glandulifera* by bumblebee workers increase with the air temperature. However, it is necessary to investigate whether revisits are errors in flower choice or determined by very fast nectar replenishment in *I. glandulifera* flowers. The first scenario would be alarming in terms of global warming because female workers provide food for larvae, while the second scenario would indicate the higher foraging efficiency of bumblebee workers than males under very high temperatures (> 37°C).

We also found that the importance of effectively feeding male bumblebees for colony success may be even higher than usually expected [67]. Although bumblebee males do not feed larvae, they participate in pupal incubation [68], and the traits of males (e.g., sperm length and quality) determine queen survival during winter as well as their longevity and reproductive success [69, 70]. If revisits of *I. glandulifera* flowers are errors in flower choice, then in very hot weather (> 37°C), males may be more efficient in foraging than female workers. On the other hand, if revisiting of probed flowers is beneficial in terms of the amount of nectar replenishment, then the role of males in a colony could be even higher; thus, at temperatures below 37°C, males will be more efficient in recognizing refilled flowers than female workers. Nevertheless, further studies are needed to verify this supposition.

## Methods

The two experiments were carried out under common garden conditions in a cultivation plot of the Institute of Nature Conservation Polish Academy of Sciences in Cracow (S Poland; plot area = 2.7 ares) using seedlings of *I. glandulifera* and *I. parviflora* transplanted from neighbouring localities. A total of 100 seedlings were used for each species, with 25 seedlings per locality. The localities for *I. glandulifera* were Marcyporęba (forest edge), Zelczyna (roadside), Tyniec (riverside) and Szczyglice (riverside), and those for *I. parviflora* were Wielkie Drogi (forest), Kraków Młynówka Królewska (green area), Kraków Rząska (forest) and Dolina Brzoskwinki (forest edge). The former species was tested in 2019 and 2020, while the latter was tested in 2020. The use of plant parts in the present study complies with international, national and institutional guidelines. In both study years, all necessary permissions were obtained for *I. glandulifera* transplantation from the wild and cultivation (permission no.: OP-I.672.2.2019.KW.2, OP-l.672.6.2020. KW) and for disturbing protected bumblebees (permission no.: OP-I.6401.97.2019.KW.2, OP-I.6401.11l.2020.MKI).

Two-year-old cold-stored (at -2°C) frigo seedlings (*N* = 104) of *Fragaria* × *ananassa* “Vibrant” (early variety) were purchased in July 2019 from a strawberry farm located ca. 30 km away from the cultivation plot. Commercial strawberries are commonly offered as cold-stored plants because they provide fresh fruits for autumn harvesting. The use of frigo seedlings in the experiments allowed for the testing of competition for pollinators by *I. glandulifera* because the flowering time of both plants overlapped. In Poland, flowering of *I. glandulifera* occurs from July to October, while the flowering of frigo strawberries could be regulated. Frigo seedlings are usually planted from mid-May to mid-July. In the experiment, planting was carried out on 11 July, and the flowering phase started 3 weeks later. In turn, the first flowers of *I. glandulifera* were recorded on 15 July. The last flowers of frigo strawberries were noted in early September, and the last survey was conducted on 30 August. The end date of *I. glandulifera* flowering was not estimated because the tested plants were eradicated after the end of the experiment.

In 2020, the same (three years old) individuals of *Fragaria* × *ananassa* were tested together with *I. parviflora*. Cold storage of seedlings was not necessary because the flowering time of both species naturally overlapped in June. In our experiment, the strawberries flowered between 10 June and 8 July. In turn, *I. parviflora* started this phase on 5 June, and as in the case of *I. glandulifera,* the plants were eradicated after the end of the experiment; in Poland, the species ended flowering in September.

The seedlings of the three studied species were cultivated in garden pots (1.1 L capacity) filled with universal garden soil mixed with sand [71, 72]. Following the methods of cultivation of *Fragaria* × *ananassa*, the substrate was additionally enriched with a fertilizer. Moreover, to protect the seedlings from Iberian slugs (*Arion lusitanicus*), the pots were wrapped with copper sticky tape. In 2019, young seedlings of *I. glandulifera* were under a high pressure exerted by this slug and nearly half of *I. glandulifera* seedlings were entirely eaten during the 10 first days of cultivation and needed to be replaced with new seedlings. It should be also noted that the eaten seedlings were short, while taller seedlings were browsed only occasionally. Although the slugs did not attack *I. parviflora* or *Fragaria* × *ananassa*, the copper sticky tape was used in all pots, regardless of plant species, completely eliminating the impact of slugs.

Individuals of two invasive alien *Impatiens* species and cultivated *Fragaria* × *ananassa* were used in two different experiments.

### Experiment 1: Invasive alien species decrease pollination of cultivated species

The aim of Experiment 1 was to check whether the alien *Impatiens* decrease pollination of cultivated strawberries. As pollinators, we define all insects visiting flowers to collect pollen or nectar and may carry pollen from the male to female flower phase (from anther to stigma).

In August 2019, *Fragaria* × *ananassa* was exposed together with the highly invasive alien *I. glandulifera*, while in June 2020, *Fragaria* × *ananassa* was exposed together with the less invasive *I. parviflora*. The former tests were performed on 10 study days (dates: 5-10.08, 12.08, 26-27.08, 30.08), while the latter were performed on 9 study days (dates: 17-19.06, 22-26.06, 29.06). Each study day pollinator activity was assessed in three variants. In ”*Impatiens* species” variant (Im), only individuals of the alien species were exposed (*I. glandulifera* or *I. parviflora*); in “*Fragaria × ananassa*” variant (Fr), only individuals of the cultivated species were exposed, while in “*Fragaria × ananassa* AND *Impatiens* species” variant (FrIm), individuals of one of the two alien species were arranged together with the cultivated ones. Each study day, the sequence of the three variants, as well as the arrangement of particular plant individuals on the plot, were randomly selected. In the Im variant, the individuals of *Fragaria* × *ananassa* were removed from the plot and closed indoors, while in Fr, the same procedure was applied for the *Impatiens* individuals. A similar number of randomly selected flowering plants per species (~30 of 100 transplanted plants) was included on each study day (the redundant flowering plants were closed indoors).

Each study day, the tests started in the morning (between 9:00 h and 11:00 h) and ended in the afternoon (between 13:30 h and 15:30 h). In each variant, the number of pollinators visiting the surveyed plants was counted over a 70-minute period. There were 30-minute breaks between the variants to ensure that pollinators recognized the change in plant species availability on the plot. In total, each study day, the observation time took 210 minutes with 60-minute breaks.

Flights of the pollinators were tracked, and ID numbers of subsequently visited plants were noted. The recorded pollinators were not caught but identified while pollinating. It should also be noted that Formicidae (Online Resource, Table S2) were not included in the analysis because they are considered to be nectar thieves rather than beneficial visitors of flowers, and their activity damages flowers and results in a significant reduction in seed set [73, 74].

The surveys were conducted in warm and windless weather. For each day and each variant, the sampling effort was similar, with the same observation time and the same researcher tracking the pollinator flights. Each study day, the number of flowers per plant was counted and the air temperature was measured using data loggers i-Button DS1921G (with 10 minute intervals). During the last survey, the height of the plants was also measured (Online Resource, Table S3).

### Experiment 2: Revisiting flowers increases with air temperature

In 2019, we also checked whether bumblebees were revisiting the same individual flowers of *I. glandulifera* that they had previously visited during the same flight and tested the factors that may determine such revisits (10 study days; dates: 5-10.08, 12.08, 26-27.08, 30.08); notably, the same *I. glandulifera* individuals were used as in Experiment 1. The assumption was that during a single flight, bumblebees would avoid flowers that were already probed and scent-marked, regardless of the nectar production potential of the plant; therefore, we did not assess the nectar content in the visited flowers. In 2019, we found that the number of revisits increased with temperature; therefore, in the next study year, we arranged a new experiment dedicated to testing bumblebee revisits under high air temperatures exceeding > 30°C. The high temperature level was estimated following the Climate of Poland 2020 report [75].

In 2020, the surveys were conducted exclusively in hot weather with air temperatures exceeding 30°C (10 study days; dates: 07-09.08, 11-14.08, 17.08, 20-21.08). As in 2019, the assessment was conducted solely with *I. glandulifera* individuals (N = 100) and their bumblebee pollinators. Likewise, in 2019, bumblebees were not caught, the assessment was carried out during their activity at flowers, and all individuals were identified to the species level. To check whether bumblebee revisits are determined by high temperature or more associated with diurnal changes in pollinator activity, we carried out surveys three times a day: in the morning (between 8:30 h and 10:30 h), at noon (between 11:30 h and 13:30 h) and in the afternoon (between 16:30 h and 18:30 h). Each survey, regardless of the time of day, took 70 minutes (210 minutes per study day). The average recorded temperatures were 28.4°C in the morning, 35.2 °C at noon and 27.3°C in the afternoon. To facilitate the quantification of revisits, before each survey in 2020, all but one representative flower per *I. glandulifera* was cut off.

Each recorded bumblebee was identified in terms of its gender/family caste (in the case of females), and its size was roughly assessed (small, average, big). The average number of flowers visited by each bumblebee individual was 9 (range: 5-32). It was estimated that a single visit to a flower took about 5 seconds; thus, each flight was monitored for approximately 45 seconds. Although bumblebee queens and female workers of *B. hypnorum* were noted in the study (N = 4 and N = 2, respectively), they were excluded from the analysis because of their numbers were too low. The temperature was measured using data loggers (with 10 minute intervals). Moreover, using a hand-held environmental metre (Extech 45170CM), sun illuminance was noted (in 35 minute intervals). Revisits of flowers may also be determined by flower size; in 2020, the area of flowers was measured using 4 flowers randomly selected per surveyed plant, which were cut off during the two last surveys, and their profiles were photographed against a millimetre paper background (Canon EOS 60D, Canon EF 100 mm f/2.8 Macro USM lens and ring flashlight). Digital images (Online Resource, Fig. S4) were analysed with ImageJ software (ver. 1.51 k). The area of one flower side was assessed (Online Resource, Table S3), which corresponded to half of the total flower area. Moreover, in 2020, we also assessed which *I. glandulifera* individuals were the first to be selected by arriving bumblebees, and we identified which flower traits determined this choice. Each year, the sampling effort during the surveys was similar, with the same observation time and the same researcher tracking the pollinator flights.

### Statistical analyses

The data were analysed with generalized linear mixed models (GLMMs) and general linear models (GLMs). The models with the lowest Akaike information (AIC) criterion (Online Resource, Table S2) were chosen [76]. Pairwise contrasts were applied for between-group comparisons.

In Experiment 1 (“Invasive alien species decrease pollination of cultivated species”), statistical analyses were carried out using SPSS ver. 26.0 [77]. The GLMM model for the analysis assumed a Poisson distribution of the number of recorded pollinators per plant individual per survey (‘N pollinators’) as a target variable (sample size = 1422). The base model (with all variables) included 4 fixed effects and two random factors (Online Resource, Table S2). Variants (Im, Fr, FrIm) and plant species (*I. glandulifera*, *I. parviflora*, *Fragaria* × *ananassa*) were combined into a single variable (‘Variant and species’), which allowed for between-species comparisons for variant FrIm, in which alien and cultivated species were exposed together. The stem height (‘Stem height’) and number of flowers (‘N flowers’) were added to the model because the three species significantly differed in these parameters (e.g., *I. glandulifera* had more flowers than the two other species and *Fragaria* × *ananassa* was very short). Although weather conditions during the surveys were consistent (warm and windless), the air temperature increased as the day progressed. Therefore, the temperature variable (‘Temperature’), which is important for pollinator activity, was also included in the model. In addition, to account for potential variation in the tested factors throughout the day, sequence of the three variants (Im, Fr, FrIm), in which pollinator activity was assessed, was assigned as ‘Time intervals’ (1st, 2nd, and 3rd) and added to the model as a random factor. Moreover, the unique ID number was assigned to each surveyed plant individual. As the number of visits per particular plant individual was different, ‘Plant ID’ was also added as a random factor. For each arrangement variant, the recorded pollinators were assigned to four broad taxa (Bombini, Syrphidae, Apoidea and Vespoidea). Differences between taxon groups recorded in Im, Fr and FrIm variants were tested separately for each plant species using Tests of Equal or Given Proportions (“proportion tests”), and the statistical analysis was carried out using R [78].

In Experiment 2 (“Revisiting of flowers increases with air temperature”), statistical analyses were carried out using R [78]. Two separate base GLMM models (with all variables) were used for each study year (Online Resource, Tables S2). The target variable, i.e., the number of revisits per flower per pollinator (‘N revisits’), was similar in both models. Because no revisits (value 0) occurred in most cases (75.6% and 90.3% of records in 2019 and 2020, respectively; zero-inflation parameter in both years < 0.05), the target variable was tested using AD Model Builder with a zero inflation parameter and a negative binomial distribution (the glmmADMB package) [79]. In the model for 2019 (sample size = 1435), the fixed effects were air temperature (‘Temperature’), bumblebee species (‘Pollinator’), and cloud cover (cloudy, sunny or partially sunny; ‘Cloud cover’). Because different bumblebee species have different tolerances to temperature (darker species are more sensitive to high temperatures), the interaction between air temperature and species was also included in the model. Moreover, the number of pollinator visits was different for individual plants; therefore, we added ‘Plant ID’ as a random effect (Online Resource, Table S2). In 2020, more variables and interactions were included in the model and the sample size was larger (*N* = 5639). The air temperature, pollinator species and ID number of plants as random effects were consistent with those used in 2019; however, cloud cover was replaced by sun illuminance measurements (‘Sun radiation’). New variables were also included: gender/family caste (male/female worker; ‘Gender’) of pollinators, their approximated size (‘Size’), time of the survey (‘Day time’) and area of flower profiles (‘Flower area’; Online Resource, Fig. S4). The interaction ‘Temperature*Pollinator’ was also included in the model. As the effect of temperature may interact with gender/caste or bumblebee size, ‘Temperature*Gender’ and ‘Temperature*Size’ were also included.

To test which individuals of *I. glandulifera* are the first to be selected by arriving bumblebees, the number of first visits in particular surveys was counted for each individual plant and used as a target variable (‘N first visits’) in the GLM model with a Poisson distribution (sample size = 244; Online Resource, Table S4). Four individuals who were never the first to be visited were also included in the model. Moreover, four fixed effects were included: ‘Stem height’, ‘Flower area’, ‘Flower hue’ and ‘Location’. Flower hue, that plays a significant role in pollination by animals [80], was included because particular individuals of *I. glandulifera* differed in this respect, having light (pinkish) or dark (reddish) flowers. We also added a binary variable – location – that allowed to test whether individuals from the plot margins were more frequently first visited than plants situated in central parts of the plot. ArcGIS geographic information system was used to graphically visualize the first visited plants (Online Resource, Fig. S3).

## Supplementary Information

**Additional file 1: Table S1**. Pollinators recorded from flowers of cultivated *Fragaria × ananassa*, highly invasive alien *Impatiens glandulifera* and invasive alien species *I. parviflora* in Experiment 1. The table also includes Formicidae, which is known to include nectar thieves. **Table S2**. Base, candidate and best-fit models in Experiments 1 and 2. The selected best-fit model (bolded) had the lowest corrected Akaike information value AICc. **Table S3**. Stem height of individuals of cultivated *Fragaria × ananassa*, highly invasive alien *Impatiens glandulifera* and invasive alien species *I. parviflora*. Data on the profile area of flowers of *I. glandulifera* (see Fig. S4) are also included. **Table S4**. GLM model testing factors correlated with an *Impatiens glandulifera* individual being the first to be chosen by visiting bumblebees. The number of first choices for each plant was a target variable, while the stem height, flower size, flower hue and location were fixed effects. **Figure S1**. Effect of air temperature on the estimated number of revisits of flowers of *Impatiens glandulifera* by bumblebees (*Bombus lucorum complex*, *B. pascuorum* and *B. terrestris*). **Figure S2**. Effect of cloudy cover on the mean number of revisits (±SE) of flowers of *Impatiens glandulifera* by bumblebees (*Bombus lucorum complex*, *B. pascuorum* and *B. terrestris*). **Figure S3**. Spatial array of *Impatiens glandulifera* individuals chosen by bumblebees as first to be visited. In each survey (S1-S10), the total number of first visits per particular plant is demonstrated using a colour scale. **Figure S4**. Profile of the *Impatiens glandulifera* flower against a millimetre paper.

## Data Availability

The data generated or analyzed during the current study are included in this published article and its supplemental data files and available from the corresponding author on reasonable request.
