# Supplementary material for: Alien balsams, strawberries and their pollinators in a warmer world
Source: BMC Plant Biol. 2021 Oct 30;21:500. doi: 10.1186/s12870-021-03282-1 (PMC8556960; doi:10.1186/s12870-021-03282-1)
Supplement: Supplementary file 1 — Additional file 1: Table S1. Pollinators recorded from flowers of cultivated Fragaria × ananassa, highly invasive alien Impatiens glandulifera and invasive alien species I. parviflora in Experiment 1. The table also includes Formicidae, which is known to include nectar thieves. Table S2. Base, candidate and best-fit models in Experiments 1 and 2. The selected best-fit model (bolded) had the lowest corrected Akaike information value AICc. Table S3. Stem height of individuals of cultivated Fragaria × ananassa, highly invasive alien Impatiens glandulifera and invasive alien species I. parviflora. Data on the profile area of flowers of I. glandulifera (see Fig. S4) are also included. Table S4. GLM model testing factors correlated with an Impatiens glandulifera individual being the first to be chosen by visiting bumblebees. The number of first choices for each plant was a target variable, while the stem height, flower size, flower hue and location were fixed effects. Figure S1. Effect of air temperature on the estimated number of revisits of flowers of Impatiens glandulifera by bumblebees (Bombus lucorum complex, B. pascuorum and B. terrestris). Figure S2. Effect of cloudy cover on the mean number of revisits (±SE) of flowers of Impatiens glandulifera by bumblebees (Bombus lucorum complex, B. pascuorum and B. terrestris). Figure S3. Spatial array of Impatiens glandulifera individuals chosen by bumblebees as first to be visited. In each survey (S1-S10), the total number of first visits per particular plant is demonstrated using a colour scale. Figure S4. Profile of the Impatiens glandulifera flower against a millimetre paper. [file 12870_2021_3282_MOESM1_ESM.pdf]

Supporting information

Title: Alien balsams, strawberries and their pollinators in a warmer world

Authors: Kamil Najberek, Andrzej Kosior, Wojciech Solarz

Email addresses: Kamil Najberek najberek@iop.krakow.pl, Andrzej Kosior kosior@iop.krakow.pl, Wojciech Solarz solarz@iop.krakow.pl

Tables

Table S1 Pollinators recorded from flowers of cultivated *Fragaria* × *ananassa*, highly invasive alien *Impatiens glandulifera* and invasive alien species *I. parviflora* in Experiment 1. The table also includes Formicidae, which is known to include nectar thieves.

Plant species	Taxon	N records
	Syrphidae	600
	Formicidae	69
	Episyrphus balteatus	24
	Apidae	16
	Bombus pascuorum	11
	Muscidae	8
	Symphyta	7
	Piophilidae	7
	Bombus lucorum -complex	7
	Eupeodes corollae	5
	Lasioglossum	5
	Empididae	5
	Bombus terrestris	3
Fragaria × ananassa	*Apis mellifera*	3
	Psithyrus vestalis	2
	Bombus humilis	2
	Tenthredinoidea	2
	Chloropidae	1
	Syrphini	1
	Buprestidae	1
	Nematocera	1
	Geotrupidae	1
	Bombus pratorum	1
	Chloropidae	1
	Calliphoridae	1
	Vespula vulgaris	1
	Vespidae	1
	**Total number of records:**	786
	Bombus lucorum-complex	1180
	Bombus pascuorum	485
	Bombus terrestris	206
	Apis mellifera	107
Impatiens glandulifera	*Vespula vulgaris*	15
	Apidae	13
	Vespidae	11
	Syrphidae	4
	Panorpa communis	1
	**Total number of records:**	2022
	Syrphidae	407
	Muscidae	6
	Episyrphus balteatus	5
	Apidae	4
	Tenthredinoidea	2
Impatiens parviflora	*Psithyrus vestalis*	2
	Formicidae	2
	Drosophilidae	1
	Sciaridae	1
	Eupeodes corollae	1
	Lasioglossum	1
	Calliphoridae	1
	**Total number of records:**	433

Table S2 Base, candidate and best-fit models in Experiments 1 and 2. The selected best-fit model (bolded) had the lowest corrected Akaike information value AICc.

Models for the number of pollinators ('N pollinators') as a target variable counted per plant individual in particular surveys (Experiment 1).

Model	AICc value
Base model:<br>N pollinators ~ Variant and species + Stem height + N flowers + Temperature + (1|Plants ID) + (1|Time intervals)	3666.15
Candidate models:<br>N pollinators ~ Variant and species + Stem height + Temperature + (1|Plants ID) + (1|Time intervals)	3659.16
N pollinators ~ Variant and species + Stem height + N flowers + (1|Plants ID) + (1|Time intervals)	3655.44
**N pollinators ~ Variant and species + Stem height + (1|Plants ID) + (1|Time intervals)**	**3648.71**

Models for the number of revisits ('N revisits') as a target variable counted during a single pollinator flight (Experiment 2).

Model	AIC value
**2019:**	
Base model:<br>N re-visits ~ Temperature + Pollinator + Cloud cover + Temperature*Pollinator + (1|Plant ID)	1977.26
Candidate models:<br>N re-visits ~ Temperature + Pollinator + Temperature*Pollinator + (1|Plant ID)	1978.40
N re-visits ~ Temperature + Pollinator + (1|Plant ID)	1976.70
**N re-visits ~ Temperature + Pollinator + Cloud cover + (1|Plant ID)**	**1975.20**
**2020:**	
Base model:<br>N re-visits ~ Temperature + Day time + Pollinator + Gender + Size + Sun radiation + Flower area + Temperature*Pollinator + Temperature*Gender + Temperature*Size + (1|Plant ID)	3795.56
Candidate models:<br>N re-visits ~ Temperature + Day time + Pollinator + Gender + Size + Sun radiation + Flower area + Temperature*Gender + (1|Plant ID)	3790.88
N re-visits ~ Temperature + Pollinator + Gender + Flower area + Temperature*Gender + (1|Plant ID)	3788.38
**N re-visits ~ Temperature + Day time + Pollinator + Gender + Flower area + Temperature*Gender + (1|Plant ID)**	**3787.00**

Table S3 Stem height of individuals of cultivated *Fragaria* × *ananassa*, highly invasive alien *Impatiens glandulifera* and invasive alien species *I. parviflora*. Data on the profile area of flowers of *I. glandulifera* (see Fig. S4) are also included.

Experiment 1

Species	N individuals	Stem height [cm]		
		Minimal	Maximal	Average
Fragaria × *ananassa*	62	7	28	12
Impatiens glandulifera	42	65	177	149
Impatiens parviflora	45	20	54	38

Experiment 2

Species	N individuals	Stem height [cm]			Flower area [mm^2]		
		Minimal	Maximal	Average	Minimal	Maximal	Average
Impatiens glandulifera	60	70	182	128	337	782	556

Table S4 GLM model testing factors correlated with an *Impatiens glandulifera* individual being the first to be chosen by visiting bumblebees. The number of first choices for each plant was a target variable, while the stem height, flower size, flower hue and location were fixed effects.

Effect	$\chi 2$	df	p
Stem height	0.84	1	0.4
Flower area	0.02	1	0.9
Flower hue	1.73	2	0.4
Location	0.004	1	0.9

Figures

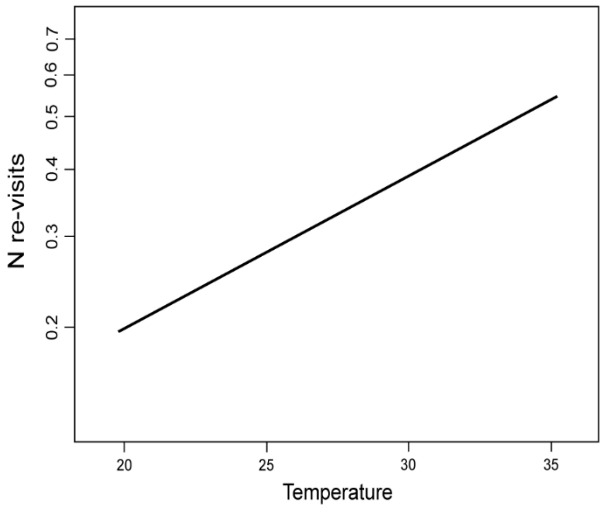

Fig. S1 Effect of air temperature on the estimated number of revisits of flowers of *Impatiens glandulifera* by bumblebees (*Bombus lucorum complex*, *B. pascuorum* and *B. terrestris*).

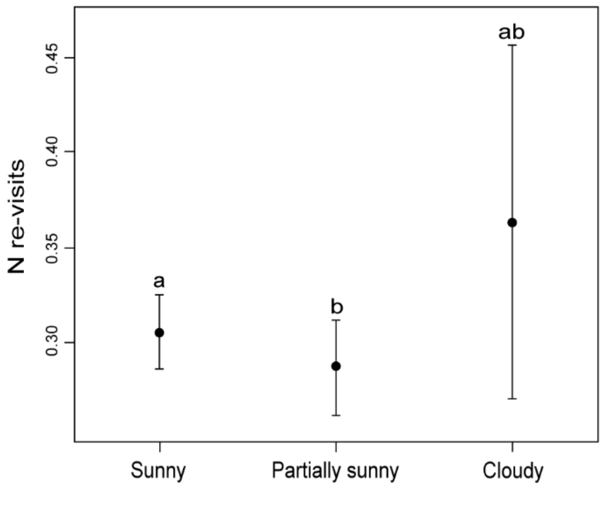

Fig. S2 Effect of cloudy cover on the mean number of revisits (±SE) of flowers of *Impatiens glandulifera* by bumblebees (*Bombus lucorum complex*, *B. pascuorum* and *B. terrestris*).

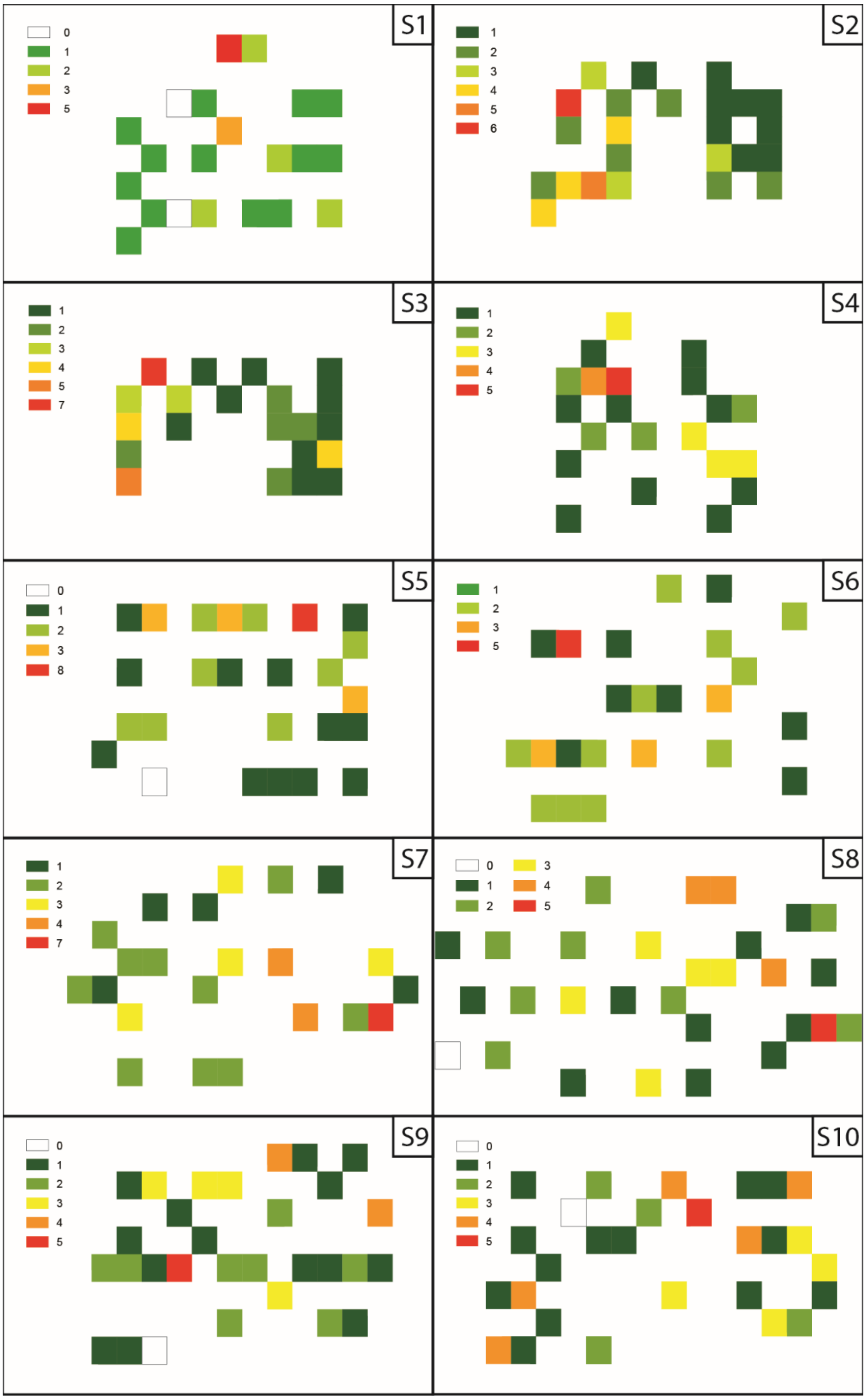

Fig. S3 Spatial array of *Impatiens glandulifera* individuals chosen by bumblebees as first to be visited. In each survey (S1-S10), the total number of first visits per particular plant is demonstrated using a colour scale.

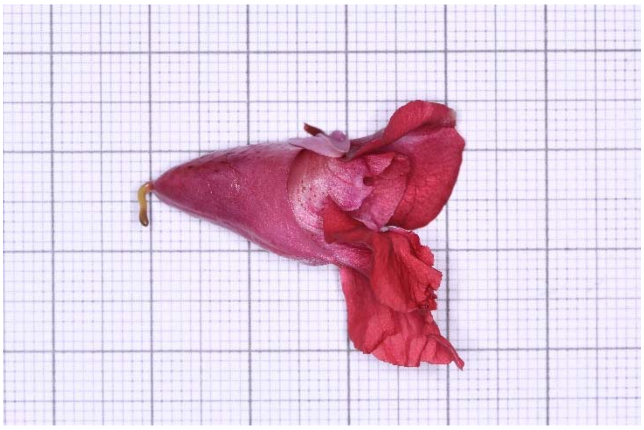

Fig. S4 Profile of the *Impatiens glandulifera* flower against a millimetre paper.
